# Predicting periprosthetic joint infection: external validation of preoperative prediction models

**DOI:** 10.5194/jbji-9-231-2024

**Published:** 2024-10-25

**Authors:** Seung-Jae Yoon, Paul C. Jutte, Alex Soriano, Ricardo Sousa, Wierd P. Zijlstra, Marjan Wouthuyzen-Bakker

**Affiliations:** 1 Department of Orthopaedic Surgery, University Medical Center Groningen, University of Groningen, Groningen, the Netherlands; 2 Infectious Diseases Service, Clínic Barcelona, University of Barcelona, Barcelona, Spain; 3 Porto Bone Infection Group (GRIP), Orthopaedic Department, Centro Hospitalar Universitário do Porto, Porto, Portugal; 4 Department of Orthopaedic Surgery, Medical Center Leeuwarden, Leeuwarden, the Netherlands; 5 Department of Medical Microbiology and Infection Prevention, University Medical Center Groningen, University of Groningen, Groningen, the Netherlands

## Abstract

**Introduction**: Prediction models for periprosthetic joint infections (PJIs) are gaining interest due to their potential to improve clinical decision-making. However, their external validity across various settings remains uncertain. This study aimed to externally validate promising preoperative PJI prediction models in a recent multinational European cohort.

**Methods**: Three preoperative PJI prediction models – by Tan et al. (2018), Del Toro et al. (2019), and Bülow et al. (2022) – that have previously demonstrated high levels of accuracy were selected for validation. A retrospective observational analysis of patients undergoing total hip arthroplasty (THA) and total knee arthroplasty (TKA) at centers in the Netherlands, Portugal, and Spain between January 2020 and December 2021 was conducted. Patient characteristics were compared between our cohort and those used to develop the models. Performance was assessed through discrimination and calibration.

**Results**: The study included 2684 patients, 60 of whom developed a PJI (2.2 %). Our cohort differed from the models' original cohorts with respect to demographic variables, procedural variables, and comorbidity prevalence. The overall accuracies of the models, measured with the c statistic, were 0.72, 0.69, and 0.72 for the Tan, Del Toro, and Bülow models, respectively. Calibration was reasonable, but the PJI risk estimates were most accurate for predicted infection risks below 3 %–4 %. The Tan model overestimated PJI risk above 4 %, whereas the Del Toro model underestimated PJI risk above 3 %.

**Conclusions**: The Tan, Del Toro, and Bülow PJI prediction models were externally validated in this multinational cohort, demonstrating potential for clinical application in identifying high-risk patients and enhancing preoperative counseling and prevention strategies.

## 1 Introduction

Periprosthetic joint infection (PJI) is a devastating complication of total hip arthroplasty (THA) and total knee arthroplasty (TKA). Treatment of a PJI involves multiple operations and prolonged antibiotic therapy, which are associated with reduced quality of life and high healthcare costs (Cahill et al., 2008; Hackett et al., 2015; Kurtz et al., 2008). With the increasing demand for arthroplasties in aging populations and PJIs being the leading cause of revision arthroplasties, the burden that PJIs place on healthcare systems is expected to rise (Premkumar et al., 2021; Dale et al., 2012; Bourne et al., 2004).

Improving and tailoring the prevention of PJIs can be facilitated by knowing individual patients' risks. There has been a growing interest in prediction models for PJI, which have the potential to improve patient counseling and clinical decision-making. However, most published PJI prediction models have not undergone external validation or have only been externally validated in one additional healthcare setting, typically in proximity to the institution at which they were developed (Kunutsor et al., 2017; Sweerts et al., 2023; Tan et al., 2018; Bülow et al., 2022). Considerable heterogeneity exists with respect to factors influencing the occurrence of PJI across institutions and countries, including patient characteristics, infection prevention practices, diagnostic criteria for PJI, and definitions of predictors (Gromov et al., 2014; Franklin et al., 2017; Paxton et al., 2019). All of these factors may cause prediction models to be inaccurate and potentially harmful in certain settings (Van Calster et al., 2023). Moreover, changes in preventive strategies over time can diminish the accuracy of models developed from outdated patient cohorts. As such, no prediction model can truly be considered externally valid unless it has been tested across multiple regions and over time (Van Calster et al., 2023).

Therefore, the aim of this study was to externally validate the most promising preoperative PJI prediction models using a recent European patient cohort undergoing THA or TKA.

## 2 Materials and methods

This external validation study was conducted and reported according to the Transparent Reporting of a multivariable prediction model for Individual Prognosis or Diagnosis (TRIPOD) statement (Collins et al., 2015).

### 2.1 Identification of prediction models

We conducted a literature search to identify all preoperative PJI prediction models for THA or TKA reported until December 2022. Of the retrieved models, those that had previously demonstrated high levels of accuracy (discrimination or calibration) were selected for validation. Models were excluded if they included predictors that were not obtainable prior to surgery, predicted the risk of surgical site infection (SSI) or recurrent PJI, demonstrated poor external validity, or did not report performance metrics. Our search revealed three promising models developed by Tan et al. (2018) (hereafter referred to as “Tan”), Del Toro et al. (2019) (hereafter referred to as “Del Toro”), and Bülow et al. (2022) (hereafter referred to as “ Bülow”), respectively. The Tan and Del Toro models were developed for both THAs and TKAs, whereas the Bülow model is only intended for primary THAs. These models showed good predictive performance in the United States (US), Spain, and Sweden, respectively. Moreover, the Tan and Bülow models were externally valid in respective cohorts from the US and Denmark. These three models were therefore regarded as having high clinical potential.

### 2.2 Study design and participants

We performed a multicenter retrospective study at four secondary- and tertiary-care hospitals in the Netherlands, Portugal, and Spain. Institutional review board approval was obtained at each hospital. All adult patients who underwent primary or aseptic revision THA or TKA in the period from January 2020 to December 2021, with a follow-up of at least 1 year, were included. The cohort with the diagnosis of aseptic revision were selected based on the postoperative diagnosis. Patients who were diagnosed with a PJI during revision surgery were excluded. As the Bülow model was developed on patients receiving primary THA, it was validated in a subset of our study population comprised solely of such patients.

### 2.3 Data collection

**Figure 1:**
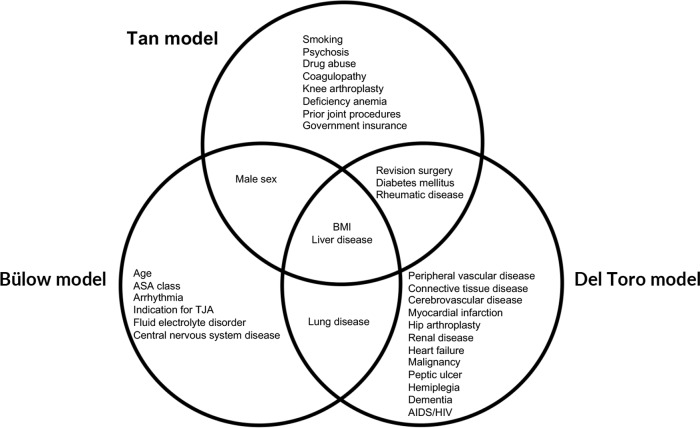
Venn diagram of the predictors included in the Tan, Del Toro, and Bülow models.

Patient charts from electronic health records were manually reviewed to collect demographic data, including age, sex, and body mass index (BMI). Procedural variables were also collected, including the affected joint, number of prior surgeries on the joint, type of arthroplasty (primary vs. revision), duration of surgery, anesthesia (regional vs. general), and principal diagnosis for arthroplasty. All comorbidities used by the Tan, Del Toro, and Bülow models were collected (Fig. 1). Comorbidities in the patient charts were assessed using the Elixhauser and Charlson comorbidity indices (Elixhauser et al., 1998; Charlson et al., 1987). If a PJI developed during follow-up, this was recorded as an event. A PJI was defined according to the European Bone and Joint Infection Society (EBJIS) criteria (McNally et al., 2021).

### 2.4 Missing data

Although the original models were developed via complete case analysis, we did not exclude patients with missing data, as this can lead to reduced power and biased estimates (Harrell et al., 1996). After ascertaining that the missing data pattern was consistent with a missing-at-random assumption, multiple imputation was performed using chained equations (White et al., 2011; van Buuren and Groothuis-Oudshoorn, 2011). A total of 20 imputed datasets were generated for this procedure. Predicted risks and performance measures were estimated in each of the 20 datasets and pooled using Rubin's rules.

### 2.5 Model validation

To determine the extent to which our cohort differed from the populations in which the models were previously studied, we performed $\chi^{2}$ tests of homogeneity using patient characteristics. Statistical significance was considered when the p value was less than 0.05.

Model performance was assessed in concordance with a suggested framework for appraising prediction models (Steyerberg et al., 2010): we evaluated discrimination through the c statistic and calibration through the calibration plot, intercept, and slope. The c statistic, also known as the area under the receiver operating characteristic curve (ROC), measures a model's ability to distinguish between patients with an event and patients without an event. The score ranges from 0.5 to 1.0, with scores closer to 1.0 indicating better discrimination (Royston and Altman, 2010). Calibration refers to how closely the predicted risks match the observed rates of the event. This can be visualized through a calibration plot, in which a perfect model has a slope of 1 and an intercept of 0 (Van Calster et al., 2016).

The Tan model was modified to exclude the “government insurance” variable from calculations, as receiving government insurance is an indicator of disease burden, age, or socioeconomic status in the US but not in countries with universal health coverage.

All analyses were performed in R version 4.2.1 (R Foundation for Statistical Computing) using the CalibrationCurves (Van Calster et al., 2016; De Cock et al., 2023), dplyr (Wickham et al., 2023), and mice packages (van Buuren and Groothuis-Oudshoorn, 2011).

## 3 Results

### 3.1 Patient characteristics

We included a total of 2684 patients in our cohort, 1528 of whom received primary THA. PJI was observed in 60 (2.2 %) patients in the entire cohort and in 33 (2.2 %) patients undergoing primary THA. The characteristics of our entire cohort and the primary THA subgroup (second and third columns) are presented alongside the characteristics of the cohorts used to develop the models (fourth to sixth columns) in Table 1.

**Table 1:** Comparison of the patient characteristics between the validation and derivation cohorts.

	Validation cohort		Derivation cohorts			p value^*^		
Patient characteristic	All patients	Primary THA	Tan	Del Toro	Bülow	Tan	Del Toro	Bülow
Number of patients	2684	1528	27 717	2324	88 830			
Inclusion period	2020–2021	2020–2021	2000–2014	2013–2015	2008–2014			
Demographic								
Age (years)	70 (62, 77)^a^	70 (61, 77)^a^	64 ± 12^b^	71 (64, 78)^a^	70–80^c^			
Female sex	1661 (61.9)	899 (58.8)	15 468 (55.8)	1601 (68.9)	50 151 (56.5)	<0.001	<0.001	0.07
BMI (kg m^-2^)	27.9 (24.8, 31.3)^a^	27.1 (24.2, 30.2)^a^	30.0 ± 10.3^b^		25.0–30.0^c^			
Procedural								
Type of prosthesis								
Primary hip	1528 (56.9)	1528 (100)	14 700 (53.0)^d^	947 (40.7)^d^	88 830 (100)	<0.001	<0.001	
Revision hip	199 (7.4)	0 (0)			0 (0)			
Primary knee	890 (33.2)	0 (0)	12 092 (43.6)^d^	1377 (59.3)^d^	0 (0)	<0.001	<0.001	
Revision knee	67 (2.5)	0 (0)			0 (0)			
Diagnosis								
Primary osteoarthritis	1917 (71.4)	1083 (70.8)			78 578 (88.5)			<0.001
Secondary osteoarthritis	403 (15.0)	241 (15.8)			5271 (5.9)			<0.001
Sequelae after childhood hip disease	41 (1.5)	36 (2.4)			1862 (2.1)			0.54
Avascular necrosis	138 (5.1)	130 (8.5)			1895 (2.1)			<0.001
Inflammatory joint disease	34 (1.3)	20 (1.3)			1224 (1.4)			0.91
Other	122 (4.5)	4 (0.3)			1895 (2.1)			<0.001
Duration of surgery								
≤120 min	1935 (72.1)	1088 (71.2)		2068 (89.0)			<0.001	
>120 min	471 (17.5)	294 (19.2)		256 (11.0)			<0.001	
PJI	60 (2.2)	33 (2.2)	1035 (3.7)	43 (1.9)	2173 (2.5)	<0.001	0.39	0.52
Comorbidities								
Charlson comorbidity index								
0	1397 (52.0)	824 (53.9)		1199 (51.6)	67 437 (75.9)			<0.001
1 or more	1287 (48.0)	704 (46.1)		1125 (48.4)	21 393 (24.0)			<0.001
ASA classification								
1	191 (7.1)	146 (9.6)			21 087 (23.7)			<0.001
2	1381 (51.5)	795 (52.0)			52 798 (59.4)			<0.001
3 or more	1096 (40.8)	586 (38.4)			14 945 (16.8)			<0.001
HIV/AIDS	12 (0.4)	10 (0.7)			16 (0.0)			<0.001
Anemia	69 (2.6)	40 (2.6)			619 (0.7)			<0.001
Arrythmia	267 (9.9)	144 (9.4)			6368 (7.2)			<0.001
Cancer	198 (7.4)	126 (8.2)			3997 (4.5)			<0.001
CNS disease	162 (6.0)	101 (6.6)			3142 (3.5)			<0.001
Coagulopathy	36 (1.3)	24 (1.6)			329 (0.4)			<0.001
Congestive heart failure	112 (4.2)	52 (3.4)		110 (4.7)			0.37	
Diabetes mellitus	371 (13.8)	162 (10.6)		544 (23.0)	5973 (6.7)		<0.001	<0.001
Drug abuse	17 (0.6)	15 (1.0)			682 (0.8)			0.42
Fluid and electrolyte disorders	35 (1.3)	20 (1.3)			534 (0.6)			<0.001
Kidney disease	182 (6.8)	111 (7.3)			883 (1.0)			<0.001
Liver disease	61 (2.3)	38 (2.5)		16 (0.7)	524 (0.6)		<0.001	<0.001
Lung and airway disease	275 (10.2)	147 (9.6)			4317 (4.9)			<0.001
Myocardial infarction	122 (4.5)	72 (4.7)			3087 (3.5)			0.01
Peptic ulcer	40 (1.5)	29 (1.9)			536 (0.6)			<0.001
Rheumatic disease	209 (7.8)	109 (7.1)			3926 (4.4)			<0.001

Data are presented as the number and percentage of patients unless otherwise indicated. Characteristics that were not reported by the authors of the models were left blank. The abbreviations used in the table are as follows: THA, total hip arthroplasty; BMI, body mass index; PJI, periprosthetic joint infection; ASA, American Society of Anesthesiologists; and CNS, central nervous system. ^a^ Median (interquartile range). ^b^ Mean ± standard deviation. ^c^ Mode interval. ^d^ The frequencies of primary and revision surgeries were grouped together by Tan et al. (2018) and Del Toro et al. (2019). ^*^ p values derive from $\chi^{2}$ tests of homogeneity between frequencies of characteristic variables in the derivation vs. validation population. Populations that had missing data were excluded from the comparison for that characteristic variable. We considered that a p value of 0.05 indicated statistical significance.

Our validation cohort for the Tan model had a significantly lower PJI rate (2.2 % vs. 3.7 %, p<0.001 ), a higher proportion of female patients (61.9 % vs. 55.8 %, p<0.001 ), and a higher rate of hip arthroplasty (64.3 % vs. 53.0 %, p<0.001 ) compared with the derivation cohort. Comparison of patient characteristics was limited to the published data.

Our Del Toro validation cohort had significantly more hip arthroplasties (64.3 % vs. 40.7 %, p<0.001 ), longer surgery durations ( p<0.001 ), and a higher prevalence of liver disease (2.3 % vs. 0.7 %, p<0.001 ) compared with the derivation cohort. On the other hand, fewer female patients (61.9 % vs. 68.9 %, p<0.001 ) and cases of diabetes mellitus (13.8 % vs. 23.0 %, p<0.001 ) were observed. The PJI rate was slightly higher in our cohort, but statistical significance was not observed ( p=0.39 ).

Our Bülow validation cohort, consisting of only primary THA patients, had higher rates of secondary osteoarthritis and avascular necrosis but a lower rate of primary osteoarthritis ( p<0.001 for all) than the derivation cohort. We also observed significantly higher Charlson comorbidity index scores ( p<0.001 ) and higher ASA classification scores ( p<0.001 ) in our validation cohort. Comorbidities were generally more prevalent in our validation cohort; however, the PJI rate did not differ significantly ( p=0.52 ).

### 3.2 Missing data

The number of patients with missing data was 411 (15 %). For the majority of the cases with missing data, only one variable was missing. The variables with the most missing data were duration of surgery, BMI, and ASA classification.

### 3.3 Distribution of predicted risks

**Figure 2:**
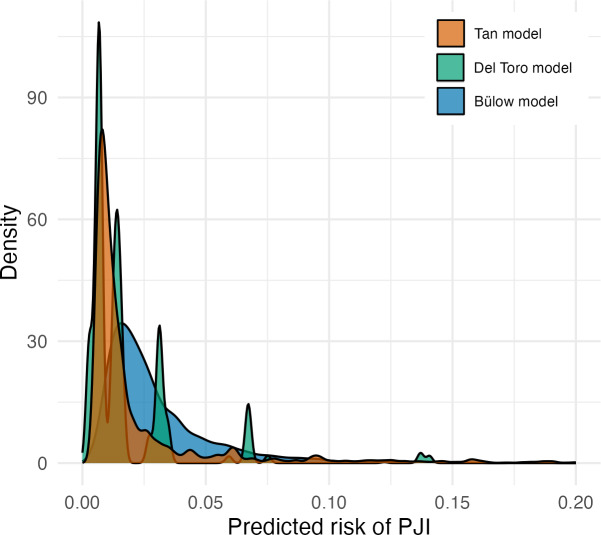
A density plot of the Tan, Del Toro, and Bülow preoperative PJI prediction models, showing the distribution of the predicted risks generated by each model.

The distribution of predicted risks from all three models were skewed to the right, with predictions above 10 % rarely observed (Fig. 2). The Tan model generated risk estimates above this value the most frequently. In contrast to the other models, the Del Toro model displayed distinct peaks in its density plot, as it can only generate 16 discrete risk estimates from its four binary variables.

### 3.4 Model performance

All three models exhibited comparable, strong discrimination, with c statistics for the Tan, Del Toro, and Bülow models of 0.72 (95 % CI: 0.65, 0.78), 0.69 (95 % CI: 0.59, 0.78), and 0.72 (95 % CI: 0.62, 0.81), respectively (Table 2).

**Table 2:** Statistical performance of the Tan, Del Toro, and Bülow preoperative PJI prediction models.

Performance statistic	Tan model	Del Toro model	Bülow model
c statistic (95 % CI)	0.72 (0.65, 0.78)	0.69 (0.59, 0.78)	0.72 (0.62, 0.81)
Calibration intercept	-0.44 ( -0.72 , -0.17 )	0.19 ( -0.07 , 0.45)	-0.35 ( -0.70 , 0.00)
Calibration slope	0.51 (0.33, 0.68)	0.74 (0.59, 0.78)	1.23 (0.76, 1.69)

**Figure 3:**
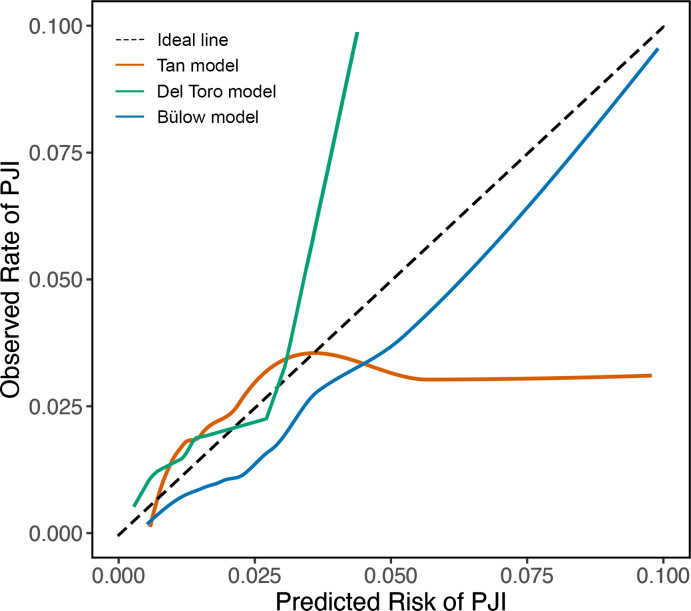
Calibration plots show the agreement between the predicted and observed risks across a range of risks. The dashed line represents perfect calibration. Solid orange, green, and blue lines represent the performance of the Tan, Del Toro, and Bülow preoperative PJI prediction models, respectively. The Tan model overestimated PJI risk above 4 %; for instance, when the model predicted a PJI risk of 5 %, the observed risk was 2.6 %. The Del Toro model underestimated PJI risk above 3 %; for example, a predicted risk of 5 % corresponded to an actual risk of above 20 %. Predicted risks above 0.10 were rarely observed; therefore, they were omitted. The Tan and Del Toro models were evaluated in patients receiving either TKA or THA, whereas the Bülow model was evaluated only in patients who received primary THA.

**Figure 4:**
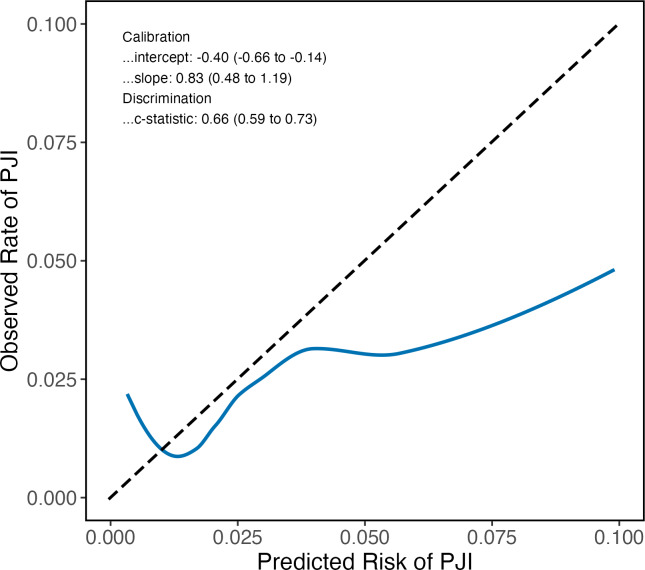
Calibration plot and performance statistics for the Bülow preoperative PJI prediction model for patients receiving THA or TKA. The dashed line represents perfect calibration. Predicted risks above 0.10 were rarely observed; therefore, they were omitted.

All models displayed reasonable calibration for predicted PJI risks below 3 %–4 %. The Tan model tended to overestimate the PJI risk above 4 %, as indicated by the calibration plot (Fig. 3), a calibration intercept of -0.44 (95 % CI: -0.72 , -0.17 ), and a calibration slope of 0.51 (95 % CI: 0.33, 0.68). For instance, when the Tan model predicted a PJI risk of 5 % for a patient, the observed risk of developing a PJI was much lower in our cohort. Conversely, the Del Toro model underestimated the PJI risk above 3 %, as shown by the calibration plot and a calibration intercept of 0.19 (95 % CI: -0.07 , 0.45). For example, patients predicted to have a 10 % risk of developing a PJI by the Del Toro model actually had a much higher PJI risk. The Bülow model generally overestimated the risk of developing a PJI, reflected by its calibration intercept of -0.35 (95 % CI: -0.70 , 0.00), but showed better calibration at higher risks than the other models. As the Bülow model was specifically developed for primary THA, we only included such procedures in our validation cohort for this model. We performed an additional analysis of the Bülow model in which we included TKA and revision THA as well, but discrimination and calibration were reduced (Fig. 4).

## 4 Discussion

The implementation of PJI prediction models is often hindered by uncertainty regarding whether they are accurate in new settings. We demonstrated the external validity of three promising preoperative PJI prediction models in a cohort that differed geographically and temporally from the cohorts in which the models were developed. All models showed good discrimination, indicating their clinical utility to identify patients that are at a higher risk of developing a PJI. However, as demonstrated by the calibration plots, the estimated risk of a patient developing a PJI was most accurate for risks up to 3 %–4 %. Although impact studies are needed, our findings demonstrate the potential of applying the three models in clinical practice as tools for risk stratification.

All three models investigated in this study exhibited performance that aligns with the upper range of performance of preoperative PJI prediction models (Kunutsor et al., 2017; Merrill et al., 2020). While certain models have demonstrated higher accuracy, they have not yet undergone external validation (Klemt et al., 2023; Yeo et al., 2023). To our knowledge, only three other preoperative PJI prediction models have been externally validated; however, their performance has been mixed. One such model, developed at an academic center in the Netherlands, showed poor discrimination ( c statistic: 0.55) and calibration during external validation at a nonacademic center (Sweerts et al., 2023, 2022). Another externally validated model, the American College of Surgeons National Surgical Quality Improvement Program Surgical Risk Calculator (ACS NSQIP SRC), initially showed excellent discrimination with a c statistic value of 0.82 (Bilimoria et al., 2013). However, lower c statistic values of 0.55, 0.71, and 0.67 were observed in other cohorts (Edelstein et al., 2015; Wingert et al., 2016; Goltz et al., 2018). Moreover, the calibration of the ACS NSQIP SRC has not been assessed, and this calculator has not been validated outside of the US. Espindola et al. (2022) derived a model that showed comparable discrimination to the Tan, Del Toro, and Bülow models; however, its full regression equation has not been published, and the model is not available as an online tool, rendering its use difficult. Considering these limitations, the Tan, Del Toro, and Bülow models emerge as favorable options in the European setting.

Our findings demonstrate the potential of the Tan, Del Toro, and Bülow models as valuable tools for risk stratification that provide accurate risk estimates in real time. These models are easy to use, requiring a small number of readily available preoperative predictors. Given their ability to classify PJI patients and uninfected patients, the use of prediction models can be beneficial for clinicians and patients during preoperative counseling and for optimization of perioperative preventive strategies. Identifying high-risk patients through prediction models presents a chance to implement additional preventive measures, such as broadening the antibiotic prophylaxis (Iannotti et al., 2020), using dual antibiotic-loaded bone cement (Jenny et al., 2021), or applying negative-pressure wound dressing (Al-Houraibi et al., 2019). Beyond clinical uses, these models hold promise for advancing precision prevention research by providing investigators with more robust methods to identify and study high-risk patients. When selecting which model to use, the advantages and disadvantages of each should be considered. The Bülow model showed the best calibration, although only for primary THA procedures; when applied to both THA and TKA, calibration was inferior to the other two models (Fig. 4). In contrast, the Tan and Del Toro models are applicable to both THA and TKA; they can classify patients above a 3 %–4 % predicted risk as having a higher risk than the general population. However, exact risks for individuals above 3 %–4 % cannot be accurately predicted. The Tan model is accessible as a mobile app and generates a more continuous range of predicted risks than the Del Toro model. Based on these considerations, we believe that the Tan model may be the most practical choice for surgeons routinely performing THA and TKA.

Our results should be interpreted in the light of several limitations. First, the retrospective nature of this study means that the accuracy and completeness of the data may be suboptimal. Second, we diagnosed PJI and comorbidities using criteria that did not precisely align with those used by the models' authors, which could have introduced biases; nevertheless, this reflects the pragmatic challenges associated with the application of these models. Third, we decided to exclude a variable from the Tan model due to its geographically dependent definition (i.e., health insurance). While this resulted in a loss of information, retaining the variable would have led to elevated predicted risks and greater overestimations. Fourth, our cohort was smaller than the derivation cohorts of two of the models, with relatively few patients developing PJI. This may have limited the precision of the performance measures for discrimination and calibration (Van Calster et al., 2016). Fifth, our study diverged from the follow-up period used by the authors of the Tan and Bülow models. We employed a minimum follow-up period of 1 year, consistent with common practice in assessing PJI risk (Xu et al., 2020) and necessary due to the recency of our cohort. This contrasted with the longer follow-up used by Tan et al. (2018), which may have captured PJI cases with a later onset that we did not observe, resulting in a significantly higher PJI rate in their cohort. Finally, the extent to which our cohort differed from the derivation cohorts could not be comprehensively assessed due to the unavailability of published data.

In conclusion, the Tan, Del Toro, and Bülow preoperative prediction models are valid tools to classify patients at high risk of PJI within Europe. These prediction models hold promise for future clinical application to intensify infection prevention measures in patients at the highest risk of developing a PJI.

## Data Availability

The code and data used in this work are available from the corresponding author upon request.
